# A statistical framework for testing the causal effects of fetal drive

**DOI:** 10.3389/fgene.2014.00464

**Published:** 2015-01-13

**Authors:** Nianjun Liu, Edward Archer, Vinodh Srinivasasainagendra, David B. Allison

**Affiliations:** ^1^Department of Biostatistics, School of Public Health, University of Alabama at BirminghamBirmingham, AL, USA; ^2^Office of Energetics, School of Public Health, University of Alabama at BirminghamBirmingham, AL, USA

**Keywords:** genetics, fetal effects, human, statistics, causal inference, intrauterine environment

## Abstract

Maternal genetic and phenotypic characteristics (e.g., metabolic and behavioral) affect both the intrauterine milieu and lifelong health trajectories of their fetuses. Yet at the same time, fetal genotype may affect processes that alter pre and postnatal maternal physiology, and the subsequent health of both fetus and mother. We refer to these latter effects as ‘fetal drive.’ If fetal genotype is driving physiologic, metabolic, and behavioral phenotypic changes in the mother, there is a possibility of differential effects with different fetal genomes inducing different long-term effects on both maternal and fetal health, mediated through intrauterine environment. This proposed mechanistic path remains largely unexamined and untested. In this study, we offer a statistical method to rigorously test this hypothesis and make causal inferences in humans by relying on the (conditional) randomization inherent in the process of meiosis. For illustration, we apply this method to a dataset from the Framingham Heart Study.

## INTRODUCTION

A common belief is that genetic, phenotypic, and behavioral characteristics of mothers affect the metabolic milieu during pregnancy, and that this in turn affects both the short and long-term metabolic health of the offspring, including the risk of obesity. This is likely true and some evidence is supportive (Catalano et al., 1998; Plagemann, 2005). Yet there is a complementary possibility that has gone largely unconsidered and untested. Namely, that the genotype of the offspring may in fact be driving (in part) the physiology and behavior of the mother, and that different fetal genotypes have differential long-term consequences on both maternal and offspring metabolic health. Using a statistical method originally developed for testing for linkage in the presence of association (Allison, 1997), we can rigorously test this hypothesis and make causal inferences, even in humans, by relying on the (conditional) randomization inherent in the process of meiosis.

The maternal–fetal relationship can and has been viewed as both one of intimate cooperation and that of intergenerational competition (Trivers, 1974; Haig, 1993; Godfray, 1995). Foundational to these views is allocation of nutrient resources to mother and fetus as well as the alteration of maternal behavior, physiology, and anatomy for the benefit or detriment of mother or fetus. From a genetic perspective, the ‘conflict of interest’ may occur because the mother’s and the fetus’ genomes are correlated, but not identical (due to the random process of meiosis and to the presence of paternal genes in the fetus; Page, 1939; Douglas et al., 1959; Haig and Westoby, 1989; Moore and Haig, 1991; Haig, 1993, 2004; Moore, 2012). As the fetus (via the placenta) gains access to the maternal blood supply, the fetus can potentially influence the behavior, physiology, and anatomy of the mother, to ‘canalize’ (Van Vleck, 1978; Gottlieb, 1991; Hallgrimsson et al., 2002) its ontogeny in a way that is well-suited to its genome. For example, a number of placental hormones manipulate maternal energy metabolism and blood supply for fetal benefit. Human placental lactogen (hPL; a diabetogenic, growth hormone-related placental hormone) plays a role in increasing maternal insulin resistance, thereby driving more energy to the fetus. Nevertheless, because hPL production is independent of maternal regulation, mothers respond to this metabolic challenge by increasing insulin production to maintain glycemic control. Additionally, increments in blood pressure increase the flux of nutrients across the intrauterine environment, and gestational hypertension induced in the first trimester leads to reduced perinatal mortality (Symonds, 1980; Hollegaard et al., 2013) and higher birth weights (Goddard et al., 2007). Nevertheless, preeclampsia (i.e., hypertension with proteinuria) is a major cause of both maternal and fetal mortality and morbidity (Haig, 1993). These results (Trivers, 1974; Haig, 1993; Godfray, 1995) suggest that fetal genes may in some cases be influencing maternal behavior, physiology, and anatomy in ways that influence the long-term health of mother, fetus, and/or both. In fact, there is recent evidence that fetal sex (a function of fetal genotype) has significant effects on maternal pre and postnatal phenotype [e.g., increased breast circumference (Galbarczyk, 2011), increased accumulation of adipose tissue, and breast milk energy content (Hinde, 2007; Powe et al., 2010; Hinde et al., 2014)]. These results and others (Takimoto et al., 1996; Kanayama et al., 2002; Tamimi et al., 2003; Dekker and Robillard, 2005; Wangler et al., 2005; Petry et al., 2007) support emerging evidence that fetal genotype affects maternal physiology both during and after pregnancy. Thus, some fetal genotypes may induce long-term effects on maternal and fetus’ postnatal health, in part via manipulation of maternal phenotype during pregnancy. Petry et al. (2007) introduced this idea, but did not offer a statistical test that could test the hypothesis and in addition, separate correlation from causation.

Identifying specific aspects of fetal genotypes (beyond the differential effects of sex chromosomes) influence maternal phenotype may be challenging for all the reasons that studying complex genetic effects are challenging in general, but also because a statistical model for testing such hypothesized effects has not yet been offered. Except in certain situations of controlled experimental crosses, observational studies showing an association between fetal genotype at a particular genomic locus and maternal phenotype merely indicate association (correlation) and not necessarily of causation. Therefore, we offer a statistical model, adapted from one developed by one of the authors in the context of family based association (i.e., ‘TDT’) testing, to rigorously test this hypothesis in humans (and other diploid populations) and make causal inferences by relying on the randomization inherent in the process of meiosis. Although the model is adapted from genetic study, the principle has its theoretic root in the causal inference literature. We describe the method here and illustrate it with data from the Framingham Heart Study (FHS).

## MATERIALS AND METHODS

### STATISTICAL MODEL

The essential proposition underlying the validity of the method we offer is that, under Mendelian theory, at any genetic locus, conditional on the parents’ mating types at that locus, the offspring’s genotype at that locus is a random variable for which the probability of each possible genotype is equal for all offspring. Therefore when we condition on parents’ mating types at a locus of interest, then we have effectively a randomized experiment in which offspring are randomly assigned to genotypes at that locus. Stated, equivalently, mothers are randomized to carry offspring of different genotypes at that locus. Hence, if we test for relations of offspring genotype with mother’s phenotype during pregnancy (e.g., preeclampsia, gestational diabetes, weight gain, or for that matter any time after conception), we can reasonably draw causal inferences about the effects of fetus genotype on mother’s pregnancy because of the aforementioned randomization, *if we condition on parental mating types at the locus under study* (Allison and Neale, 2001; Tiwari et al., 2008). Note that this causal inference refers to the effect of the fetus genotype, not necessarily the fetus’ genotype at the locus being used in the analysis. More specifically, the causal effect identified may be due to the fetal genotype at the locus under study or to the fetal genotype at another locus physically linked to and in disequilibrium with the locus under study. By this reasoning, the sex of offspring is also randomized in mammals, thus the finding that women carrying male fetuses had higher rates of gestational diabetes mellitus, indicates that having a fetus with a Y chromosome causes changes in mother’s physiology.

The above principle has been formalized in the literature of causal inference (Rubin, 1974, 1977; Hernán and Robins, 2006; Greenland and Robins, 2009). Using Rubin’s (1977) language and notation, if the experimental units are assigned into two treatment groups solely on the basis of a covariate, *X*, and random factors then causal inference can be made and in addition, causal effects of treatments can be estimated without bias. The assignment to treatment group means that if two units have the same value of *X*, then they either must receive the same treatment or must be randomly assigned to treatments (not necessarily with the same probability). The critical point is that the probability that a study unit is assigned to one treatment rather than others is a function only of the values of *X* in the sample and purely random factors. Here, *X* can either be univariate or multivariate. The principle has been further explored for observational data explicitly in Hernán and Robins (2006). They argued that in ideal randomized experiments, causal inference can be made because the randomization ensures that the exposed and unexposed are exchangeable; whereas in observational studies, because the exposed and the unexposed are not generally exchangeable therefore causal inference cannot be generally made. They reviewed a condition that permits causal inference from observational studies, that is, the conditional randomization which guarantees conditional exchangeability. These works lay a theoretical foundation for our method. Specifically, here the study unit is parents–child trio; the parental mating types which are functions of the two parental genotypes, (g_F_,g_M_), are the *X* in Rubin’s (1977) paper and the covariate used to assign study units to treatment groups; the genotype of children, g_k_, is the treatment although we have three groups here (i.e., three types of genotype); mother’s phenotype is the dependent variable *Y* using Rubin’s notation. Therefore conditional on parents’ mating types at a locus, then the offspring are randomly assigned to genotypes at that locus. Equivalently, the study unit, the parents–child trio, is randomly assigned to the genotypes of offspring solely based on the parental mating types. Based on the aforementioned previous work, causal inference can be made on the effect of offspring’s genotype (i.e., treatment effect in Rubin’s words) on mother’s phenotype (i.e., the dependent variable *Y*). Note that the causal inference is to ‘treatment assignment’ which in this case is the offspring’s genotype at the locus under study and/or the other loci physically linked to and correlated (in disequilibrium) with it (Allison, 1997). This is analogous to a randomized trial of a treatment (e.g., a diet involving eating more meat) in which strictly speaking the causal inference is about treatment assignment rather than meat *per se* because one cannot separate the effect of meat *per se* from other factors which may covary with assignment to more meat consumption such as the need to chew more. This is the reason why the class of genetic tests we are relying on are referred to as transmission *disequilibrium* tests.

Rubin (1977) discussed two general methods to estimate the causal effect. Because our primary interest is hypothesis testing, we choose to use a linear model as follows:

$$Y=\beta_{0}+X\beta_{1}+\beta_{k}g_{k}+\beta_{m}m+\varepsilon$$

where *Y* is mother’s phenotype during pregnancy, ***X*** is a matrix for covariates including environmental factors, g_k_ is genotype of fetus, m is the parental mating types at the same locus, ε is random error. In order to test the hypothesis, we only need to test H_0_ : β_k_ = 0. Note that the genotypes should be treated as categorical in the model (Rubin, 1977; Allison, 1997) so the test of null hypothesis is a two degrees of freedom test. Here, *Y* is assumed to be continuous. The same principle can be used when *Y* is other type of data (e.g., categorical, count, or survival), by use of a link function (i.e., in a generalized linear model framework; Nelder and Baker, 2004). Note also that the causal inference about the effects of fetal genotype on the mother’s pregnancy is based on the randomization achieved by conditioning on parental mating types at the locus under study, resulting in the inclusion of mating types m in the model (Rubin, 1977). Given the above principle, from the point of view of genetic study, the statistical model is in spirit the same as the method proposed by one of the authors (Allison, 1997), for testing such hypotheses. This work can be seen as an application of a previously validated and analytically derived method (Model Q5) to a novel context (Allison, 1997; Ewens et al., 2008; Of course, it is also an application of the work of Rubin, 1977). Given that the theoretical work in the literature and the extensive simulation studies have already demonstrated both the validity of concept and the performance of the model (Allison, 1997) we will not perform the simulations here. The analysis of data below is for illustration of this novel application, rather than a test of the previously established method (Allison, 1997), nor an attempt to draw biological conclusions from the small sample dataset.

### DATA

For illustration, we applied the concept and the model to a dataset from the FHS. The study began in 1948 with 5,209 adult subjects (i.e., the first generation) from Framingham, Massachusetts, and is now on its third generation of participants. The Offspring Study (i.e., the second generation) was initiated in 1971. A sample of 5,124 men and women, consisting of the offspring of the Original Cohort and their spouses was recruited. The recruitment of the third generation participants who had at least one parent in the Offspring Study and would be at least 20 years old by the close of the first exam cycle was started in 2001. A recruitment target of 4,095 Gen III participants was achieved by July of 2005. In order to draw causal inference using the model as described above, we need information from both parents and offspring (although extensions to allow for missing data from one parent with substitution of sibling data are possible (Dudbridge, 2008)), and most importantly, measurements during mothers’ pregnancy. Because there is little information about pregnancy for the original cohort, we had to use only the offspring generation (i.e., the second generation) and their offspring (the third generation). We focus on metabolic related measurements during pregnancy, specifically, gestational hypertension, diabetes, and weight gain which are all available in FHS. We chose to focus our examination on maternal metabolic function because the large variability of maternal phenotypic in response to the metabolic sequelae of pregnancy may be influenced by fetal genotypes. Gestational hypertension and diabetes are binary. For weight gain, there is only information about gaining more than 30 pounds during pregnancy or not in FHS. We need information (including genotype information) from both parents and children. After applying all the criteria, there are 109 families left, with a total of 282 children, clearly a small sample, but suitable for illustrative purposes. In the data, there are four observations of gestational hypertension, no observation of gestational diabetes, and 49 observations of weight gain in excess of 30 pounds. In the following analysis, we only use the first child in each family to avoid the complexities introduced by potential correlations among siblings.

Although information on gestational hypertension, diabetes, and weight gain is available in FHS, in the final dataset we obtained, the number of observations of hypertension and diabetes are small. Therefore we only focus on weight gain, which is binary with value 1 if the mother reported to have >30 pounds weight gain during pregnancy and 0 otherwise, that is:

y=I (had⁢ over⁢ 30⁢ lbs⁢ of⁢ weight⁢ gain)

where I( ) is the indicator function.

FTO is a gene located in chromosome region 16q12.2. Studies have revealed association of single nucleotide polymorphisms (SNPs) in this gene with obesity. The genotype we tested are four SNPs (i.e., rs9930506, rs9939609, rs1121980, and rs8050136) in FTO gene which have been shown to be associated with obesity (Dina et al., 2007; Frayling et al., 2007; Price et al., 2008; Li et al., 2010; Liu et al., 2013). We performed a test of Hardy–Weinberg equilibrium and no significant departure was found.

## RESULTS

To analyze the data, we used logistic regression. We performed analyses including and excluding mother’s age at children’s birth in the model. For the analyses we conducted, none of the mother’s age at children’s birth was significant (at α = 0.05). Therefore they were not included in the final models. **Table 1** depicts the results from the logistic regression for SNP rs9930506. The results from additional analyses are summarized in **Table 2**. *P*-values for the genotypes are from two degrees of freedom test of the two dummy variables for each genotype. Neither of the results is significant at α level of 0.05. This may indicate that the variants of these four SNPs carried by children do not have effect on mothers’ weight gain during pregnancy. Of course, given the small sample size, there is a possibility that there is not enough power to detect such effect. Given that this is an illustration of the application of a previously validated method, and the small sample size, we did not intend to arrive at any biological conclusions from this analysis.

**Table 1:** Results from logistic regression for SNP rs9930506 from data with only the first child in each family.

Independent variable	Intercept	Fetal genotype		Mating type				
Coefficient	–1.504	–0.049	0.239	0.177	–0.527	0.200	0.860	0.133
*P*-value	0.054	0.928		0.974				

*P-values for the genotypes are from two degrees of freedom test.*

**Table 2:** *P*-values of children’s genotypes from the analyses with only the first child in each family.

Statistical model	SNPs			
	rs9930506	rs9939609	rs1121980	rs8050136
Logistic regression	0.928	0.946	0.944	0.965

*P-values for the genotypes are from two degrees of freedom test.*

## DISCUSSION

The hypothesis that the fetal genotype affects maternal physiology and behavior represents a complementary view about not only some gestational diseases (e.g., pregnancy-induced hypertension and gestational diabetes) of mothers, but also long-term effects on metabolic health of both mother and offspring. In addition, the effects of this process may be cyclic over generations (Petry et al., 2007). This hypothesis does not contradict the common belief that association between fetal growth and diseases in pregnancy results from effects of the mother’s genotype and/or environment on her physiology which subsequently affect the fetus. Rather, both of these causal paths can, in principle, be active.

Both animal studies and human studies have shown the effect of fetal genotype on mother’s physiology during pregnancy (Galbarczyk, 2011; Hinde et al., 2014). This provides indirect evidence for our hypothesis. However, the direct test of this hypothesis is very difficult, especially in human studies. We illustrate that the process of meiosis provides a natural randomization which can be used in statistical analysis for causal inference and offer a simple statistical model for such analysis. This statistical model can be viewed either as stemming from causal inference literature or stemming from our previous work in genetic study. Of course, the theoretical work in causal inference provides foundation and our previous work in genetic study provides intuition and specific model (Model Q5 in Allison, 1997). Although Model Q5 proposed by Allison (1997) is conditional on three mating types excluding families with both homozygous parents, this can actually be released, leading to the more general model as proposed in this work. Allison’s (1997) Q5 model excludes families with both parents homozygous because such families add no information to the estimation of the elements of β. However, under the homoscedasticity assumption, they do contribute to the precision of the estimation of residual variance and therefore can be included, and their inclusion should result in slightly greater power under the alternative hypothesis. From the point of view of causal inference (Rubin, 1977), in principle causal inference can be made when conditional on parental mating types, given that we do not consider parent-of-origin effects (that is we assume that alleles in offspring have the same effects regardless of which parent those alleles were inherited from), because the probability of offspring genotypes can be fully determined by parental mating types. Therefore in our model we conditional on parental mating types. By using this analytic method, if a significant finding is obtained, one can justifiably claim that either the fetal genotype at the locus in question or a genotype physically linked to it has a causal effect on the maternal phenotype. It should be noted that many factors may contribute to the maternal phenotype. Our model only tests if fetal genotype is one of the factors, and if it is, it is a causal factor. To illustrate the principle, we analyzed a dataset from FHS using the model we proposed.

It should be noted that random mating is not assumed for our model. The model is based on the randomization of offspring via the random assignment to genotypes at the locus under study. The randomization holds *if we condition on parental mating types at that locus* under Mendelian theory (Allison and Neale, 2001; Tiwari et al., 2008). The inclusion of the mating types in the model is necessary for this “conditioning” to achieve the randomization, as pointed out by Rubin (1977). Because the fetal genotype is derived from parental genotypes, they are highly correlated. This may raise the concerns related to collinearity in numerical computation. However, collinearity is caused by high linear correlations between independent variables in the model. Although fetal genotype is correlated with parental genotypes, the linear correlations via Pearson correlation coefficients suggest that they are not prohibitively high. In our model, we treat the offspring’s genotypes and parental mating types as categorical so dummy variables are created for each of them. Therefore the correlation between parental mating types and fetal genotypes is modest. In the data we analyzed above, the correlation coefficients are less than 0.474 between fetal genotype and paternal mating types for the four SNPs, respectively. These low to moderate correlations may not induce undue issues of collinearity. Nevertheless, we recommend investigators assessing collinearity in their datasets and interpreting results accordingly.

Our model is based on a linear model (more general, generalized linear model) and assumes samples with unrelated study units. The model can be extended to handle data with related study units, such as biological siblings, by the use of a linear mixed model (or more general, generalized linear mixed model). The correlation between the related individuals can be dealt with via a random effect by using a matrix of kinship coefficients (a kinship coefficient is a measure of degree of genetic correlation between two individuals; Yu et al., 2006; Kang et al., 2010; Liu et al., 2011). In this work, we only use a linear model to test the fetal drive effects. Causation can be inferred because of the conditional randomization in the process of meiosis. More complicated models, such as structural equations models, may capture more about the complexity of the data and may work more efficiently. However, we focus on causal inference; therefore care must be taken in order to test causation with more complicated models. This is beyond the scope of this work and is a good topic for future research.

There are no significant findings in our data analysis. This may be because that the four SNPs we tested did not have deleterious effects on maternal metabolic function, or our analysis lacked the requisite power to detect an effect. Statistical power is influenced by many factors including effect size and sample size. Metabolic function is a complex phenotype, and we did not expect a large effect size for any of its risk factors. We only tested four SNPs in FTO gene due to its relationship with obesity, in a small sample, and think that a genome-wide scan in a much larger sample is warranted for more reliable inferences.

Although our statistical method makes the direct test of our hypothesis possible in human studies, it should be noted that conducting such studies necessitate a great deal of time and effort because both parents and offspring need to be included in the study and relevant physiological measurements must be made in the mothers during pregnancy. The effort is worthwhile because if the hypothesis is validated, our understanding of some gestational metabolic conditions may be shifted to a new level. New strategies may be developed to prevent and reduce morbidity and possibly mortality in both the mother and offspring and their future descendants.

## Conflict of Interest Statement

The authors declare that the research was conducted in the absence of any commercial or financial relationships that could be construed as a potential conflict of interest.
